# Assessment of Circulating Immune Complexes During Natural and Experimental Canine Leishmaniasis

**DOI:** 10.3389/fvets.2020.00273

**Published:** 2020-05-19

**Authors:** Manuela Gizzarelli, Eleonora Fiorentino, Nour El Houda Ben Fayala, Serena Montagnaro, Raquel Torras, Luigi Gradoni, Gaetano Oliva, Valentina Foglia Manzillo

**Affiliations:** ^1^Department of Veterinary Medicine and Animal Production, University of Naples Federico II, Naples, Italy; ^2^Unit of Vector-Borne Diseases, Department of Infectious Diseases, Istituto Superiore di Sanità, Rome, Italy; ^3^ISOQUINEM SL, Sant Feliu de Codines, Spain

**Keywords:** dog, leishmaniasis, immune complex, natural infection, experimental infection

## Abstract

Canine leishmaniasis (CanL) is a disease characterized by a large variety of clinical alterations, the majority of which being due to immune mediated mechanisms. Sick dogs usually produce high levels of *Leishmania*-specific immunoglobulins which may give rise to circulating immune complexes (CICs) whose defective clearance by scavenging macrophages induces vasculitis and their deposition in specific organs. The aim of this study was to assess the serum level of CICs in dogs exposed to natural and experimental infection. Fifty-two sera were examined, belonging to untreated groups of naïve beagles previously studied to assess the performance of anti-leishmanial vaccines under natural (no. 22 dogs) or experimental (no. 30 dogs) transmission. Sera were classified in five groups according to the dog's health condition, IFAT titer, and the bone marrow (BM) nested (n)-PCR result. A: no.10 healthy dogs before the experimental infection; B: no.10 clinically healthy dogs infected experimentally, IFAT negative (= reciprocal titer <160) and n-PCR positive; C: no.10 clinically healthy dogs naturally infected, IFAT positive at titers 160–320 and n-PCR negative; D: no.10 sick dogs experimentally infected, IFAT positive at titer >320 and n-PCR positive; E: no.12 sick dogs naturally infected, IFAT positive at titer >320 and n-PCR positive. CICs levels were assessed by ELISA method (canine CIC assay—Cloude-Clone Corporation, USA). The two groups characterized by negative IFAT (A and B) had the lowest median level of CICs (16.09 and 12.78 μg/ml, respectively). CICs value increased progressively in the group C and reached the highest levels in the groups D and E, both characterized by high antibodies titer and severe disease, independently from the mode of infection. Significant differences in CICs concentration (*p* < 0.0001) were demonstrated between A, B, and C groups when compared with D or E groups of dogs. No differences were found inside the first three groups, while differences were recorded between the last two groups of sick dogs. CICs serum concentration increased with the progress of leishmaniasis, being significantly correlated with the increase of specific antibodies over time. High CICs levels detectable by commercial ELISA proved specific to an established *Leishmania* infection in dogs in the absence of other concomitant infections, as demonstrated by the similar trend assessed in experimentally and naturally infected dogs.

**Graphical Abstract:**
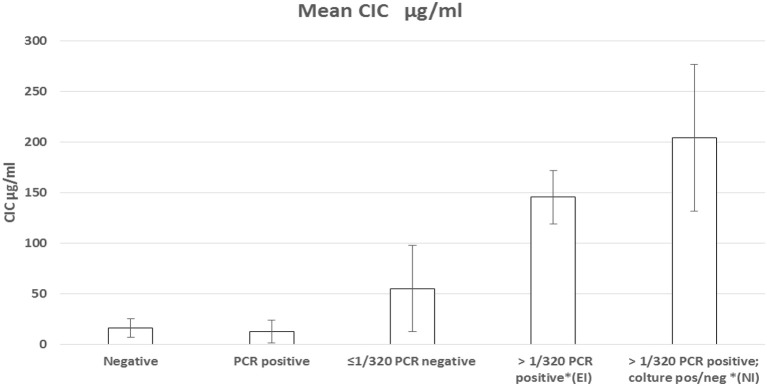
Graphical representation of CICs serum level.

## Introduction

Canine leishmaniasis (CanL) is a parasitic infection caused by the protozoan *Leishmania infantum* (Kinetoplastida: Trypanosomatidae). Affecting several millions of dogs globally (1), CanL is most often manifested as a chronic systemic disease characterized by a large variety of clinical signs and clinicopathological alterations, the majority of which due to immune mediated mechanisms. The disease progression depends largely on the immune responses mounted by infected dogs. Animals presenting overt clinical signs exhibit high titers of anti-leishmanial antibodies associated with reduced immune cellular response (2). In diseased dogs, T lymphocytes undergo depletion in the lymphoid tissues where mainly B-cell, histiocytes and macrophages proliferate, which may contribute to cause generalized lymph node enlargement, splenomegaly and hypergammaglobulinemia (2, 3). The uncontrolled concentration of antibodies and the large amount of *Leishmania* antigens can give rise to circulating immune complexes (CICs) that determine the reduction of the macrophage ability to kill the parasite and induce vasculitis that activates the complement cascade, which eventually is responsible for tissue necrosis and for some of dermal, visceral, ocular and renal lesions (2, 4, 5). Deposition of CICs in specific organs, determined by deficient activity by scavenger macrophages, results in glomerulonephritis, vasculitis, uveitis, myositis, and polyarthritis. As regards the pathogenesis of other canine vector-borne diseases (CVBDs) characterized by a progressive course of infection, the role of CICs is also well-described in different stages of infection by *Ehrlichia canis* (6), whereas this is under discussion in stages of *Anaplasma canis* infection (7–9).

Several commercial tests have been developed to detect and measure CICs from serum samples (10), that exploit different biochemical and biophysical properties such as precipitation, binding to complement fractions or Fc-recognizing molecules, however no standard tests are currently available for dogs, nor for detection of CICs during CVBDs.

The aims of the present study were to measure the serum level of CICs in dogs exposed to CanL infection, both in natural and in experimental conditions, and to assess the usefulness of a commercial ELISA kit for canine CICs detection. The justification for the use of data deriving from experimentally infected dogs is that the course of CanL infection is different when this is caused by deliberate parasite injection or laboratory-controlled sand fly bites, as compared with natural exposure to field conditions in endemic settings (1, 11). On the other hand, pathological manifestations deriving from experimental *Leishmania* infection can only be attributed to the parasite infection alone, due to the use of naïve dogs, bred under vector-borne infection-free condition in a well-controlled environment.

## Materials and Methods

A retrospective study was designed to assess the CICs level in five different groups of naïve beagles (total number: 52). These dogs belonged to untreated control groups previously studied to assess the performance of anti-leishmanial vaccines under natural (no. 22) or experimental (no. 30) transmission conditions. Field studies were performed in Italy during the years 2010–2013 and had been approved by the Veterinary Board of the Italian Ministry of Health (12). The experimental study was performed in Spain (years 2016–2017) and approved by Health Catalan Authorities (Ethical Committee authorization no. 9099).

### Natural *Leishmania* Infection

Dogs were exposed to natural conditions of *Leishmania* transmission in a rural site of southern Italy endemic for CanL, as previously reported (12). The activity of the local sand fly vector, *Phlebotomus perniciosus*, was typically seasonal (May-October); to ensure exposure to bites, the animals were not treated with repellent products with proven activity against sand flies, whereas tick and flea infestations were avoided by appropriate environmental and mechanical control measures. The dogs received routine vaccinations and were submitted to deworming every 6 months. Starting from the first month of exposure during summer, a follow-up period of 24 months was considered necessary because of the long pre-patent and incubation periods as well as the natural slow course of naturally-acquired *Leishmania* infections. This particular condition allowed to select dogs with chronic infections, most often without evident clinical disease. Dogs were examined and sampled monthly for the detection of clinical signs and clinicopathological alterations, and every 3 months for the laboratory detection and evaluation of infection.

### Experimental *Leishmania* Infection

Beagle dogs submitted to *Leishmania* experimental infection were housed at ISOQUIMEN S.L. (St. Feliu de Codres, Spain). The animals were bred under controlled conditions aimed at preventing vector-borne infections, including leishmaniasis, by mechanical measures. All dogs were under constant veterinary care, received their routine vaccinations and periodical anthelmintic treatment. The inoculum for the experimental *L. infantum* infection was prepared at the Section of Parasitology, Faculty of Farmacy of Barcelona University (Dr. Montserrat Gállego Culleré). The laboratory strain MCRI/ES/2016/BCN-890 was obtained through passage to hamster of the canine strain MCAN/ES/1992/BCN-83 (zymodeme MON-1). Parasites cultured from heavily infected hamster's spleen were used. The infection was performed by intravenous injection of recently-transformed promastigotes at the dose of 5 × 10^7^ in 1 ml physiologic saline solution. In contrast to what happens in natural *Leishmania* infections, experimentally-infected dogs usually develop infections already detectable a few weeks after receiving an intravenous injection of parasites, followed by rapid development of disease signs in most of the infected dogs (1). Dogs were examined and sampled monthly for detection of early clinical signs and clinicopathological alterations, and every 3 months for the evaluation of the infection burden.

### Serological and Parasitological Diagnosis

An in-house quantitative IFAT assay was used to detect and provide titration of anti-leishmanial immunoglobulins G, according to the technical recommendations of the Office International des Epizooties (13). Threshold for positivity (cut-off) had been previously determined at the serum dilution of 1:160 (14). Bone marrow (BM) aspirates were subject to nested-PCR (n-PCR) analysis for *Leishmania* spp. DNA (15) using the sets of R221/R332 and R223/R333 primers in two consecutive runs (16).

### Group Composition for CICs Level Assessment

Fifty-two sera stored at −80°C were examined for the detection of CICs level. Sera were classified in 5 groups according to the dog's health condition, IFAT result and titer, and the bone marrow (BM) nested (n)-PCR result. A: no.10 healthy dogs before the experimental infection; B: no.10 clinically healthy dogs infected experimentally, IFAT negative (= reciprocal titer <160) and n-PCR positive; C: no.10 clinically healthy dogs naturally infected, IFAT positive at titers 160–320 and n-PCR negative; D: no.10 sick dogs experimentally infected, IFAT positive at titer >320 and n-PCR positive; E: no.12 sick dogs naturally infected, IFAT positive at titer >320 and n-PCR positive. Because the longitudinal feature of these studies allowed to follow the dogs from their negative status through the detection of the infection till the appearance of clinical signs, the selected sera belonged to dogs with different monthly (M) periods of follow-up, respectively: Group A: M0; Group B: M3 (infection); Group C: M19 (infection); Group D: M6 (disease); Group E: M16 (disease).

CICs levels (μg/ml) were assessed in duplicate by ELISA method (canine CIC assay—Cloude-Clone Corporation, USA), according to the manufacturer's instructions. The kit consists of a competitive inhibition enzyme immunoassay technique for the *in vitro* quantitative measurement of CICs in dog serum, plasma and other biological fluids, made possible by pre-coating ELISA test plates with a canine-specific mAb. The resulting competition between biotin-labeled (standard) and unlabeled CICs (sample) is revealed and measured by horseradish peroxidase-conjugated avidin, taking into account that the color intensity developed after substrate addition is reverse proportional to the CIC concentration in the sample.

Statistical analysis was performed with MedCalc software (Frank Shoonjans, V.7.2.1.0) by the Tukey's multiple comparison test.

## Results

**Figure 1:**
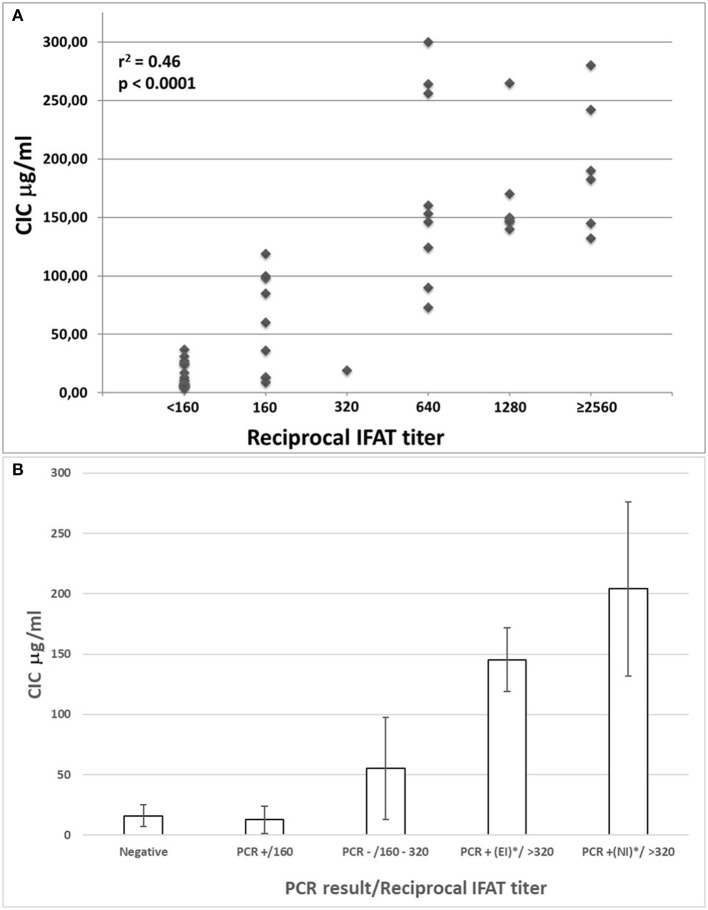
**(A)** Correlation between IFAT titers and CICs levels in sera from 52 non-infected or *Leishmania infantum*-infected beagles; **(B)** CICs serum levels (median ± SD) in five groups of beagles classified by negative condition or by *Leishmania infantum* infection characterized by different serological and parasitological parameters. EI, experimental infection; NI, natural infection.

Individual CICs and IFAT values determined in sera from all dogs were plotted and analyzed for a relationship (Figure 1A). A statistical correlation was assessed by R-squared method, resulting in a significant positive correlation explaining about half of the increased CICs values associated with elevation of IFAT titers (*r*^2^ 0.46: moderate correlation; *p* < 0.0001).

The median levels of CICs associated to parasitological and antibody responses of each of the A-E groups, are shown in Table 1. In the same table, the type and frequency of clinical and clinicopathological findings recorded in the two symptomatic groups, D and E, are also shown. Graphical Abstract of CICs levels by dog group is shown in the graph of Figure 1B. The values of single parameters referred to each dog are supplied in a separate file.

**Table 1:** CICs serum levels (median ± SD) in *Leishmania infantum* negative (Group A), infected (Groups B and C), and diseased (Groups D and E) dogs, as classified by serological (IFAT) and parasitological (bone-marrow nested-PCR) parameters, and clinical findings (proportion of dogs that expressed the alterations).

**Group (no dogs)**	**Infection**	**CICs μg/ml**	**IFAT**	**BM n-PCR**	**Lymph node enlargement**	**Weight loss**	**Anemia**	**Decreases in platelets**	**Increased BUN**	**Increased serum protein**	**A/G inversion**	**Proteinuria**
A (10)	Naïve NoI	16,09 ± 8,94	Neg	Neg	_	_	_	_	_	_	_	_
B (10)	EI	12,78 ± 11,48	Neg	Pos	_	_	_	_	_	_	_	_
C (10)	NI	55,21 ± 42,41	≤1/320	Neg	_	_	_	_	_	_	_	_
D (10)	EI	145,4 ± 26,31	>1/320	Pos	1/10	3/10	2/10	2/10	_	_	6/10	3/10
E (12)	NI	204,16 ± 72,27	>1/320	Pos	3/12	3/12	6/12	4/12	1/12	3/12	5/12	3/12

*NoI, no infection; EI, experimental infection; NI, natural infection*.

The two groups characterized by negative IFAT (A and B) had the lowest median level of CICs (16.09 and 12.78 μg/ml, respectively). Median CICs values increased progressively in the group C and reached the highest levels in the groups D and E, both characterized by high anti-leishmanial antibodies titer and severe disease, regardless of the mode of *Leishmania* infection. Significant differences in CICs concentration were demonstrated between A, B and C groups when compared with D and E groups (*p* < 0.0001). Whereas, no differences were recorded within the first three groups, CICs concentration differed significantly between D and E dogs (*p* = 0.0002). In these groups of symptomatic dogs, clinical examination revealed typical signs related to CanL such as lymph node enlargement, apathy and mild weight loss, along with frequent clinicopathological alterations in most of the dogs, such as normocytic normochromic anemia, thrombocytopenia, hyperproteinemia, reduction of serum albumin/globulin ratio. Proteinuria, as assessed by urinary protein/creatinine ratio, was found in 3/10 and 3/12 dogs of groups D and E, respectively. However, as shown in Table 1, not all dogs of groups D and E exhibited the same clinical signs and clinicopathological alterations, despite an elevated background level of CICs.

## Discussion

In several infectious diseases of humans and animals, especially those characterized by a prolonged chronic phase, the formation of CICs is promoted as a deleterious side effect of the humoral immune responses, and can cause glomerulonephritis, polyarthritis or uveitis (17). Clinical manifestations of CanL also result from both, a direct intracellular parasite activity and the dog immune response associated with deposition of CICs in different body compartments (18). Immune-mediated mechanisms play a pivotal role in the pathogenesis of many clinical manifestations of CanL, particularly in the development of four principal types of glomerulonephritis, characterized by different grade of proteinuria and responsible for the development of renal failure, the most severe complication of CanL. Previous studies found a significant correlation between the level of antibody titers when assessed by IFAT and the severity of the disease (18–20), while a recent paper (21) demonstrated that CICs concentration is clearly related to the progression of CanL in naturally-infected dogs. In this study, remarkable was the demonstration of larger-size CICs (ranging from 100 to 400 nm) formed during the worsening of the disease, which was also characterized by an increase of IFAT titers. In our study too there was a clear correlation between the CICs and IFAT values, with CICs concentration being low in clinically healthy infected dogs with undetectable or borderline anti-leishmanial antibodies. The comparison of CICs levels between the two groups of sick animals revealed that sera from naturally-infected dogs were characterized by significantly higher CICs than those from experimental infections, despite no significant differences were found in both IFAT value and clinical outcome. This could be explained by the different time course necessary to express overt clinical signs and clinicopathological alterations by the two modes of infection. As described in a previous study (12), any non-resistant *L. infantum*–infected dogs show a similar slow progress of disease during 2 years post infection, with the first detection of early clinical and clinicopathological signs in the range of 6–12 months from the exposure to natural sand fly bites. In the present study, naturally sick animals showed severe clinical manifestations of CanL in a median time of 16 months, compared with the experimentally sick dogs that manifested the disease in median period of 6 months. This difference may be attributed to the chronic stimulation of immune system caused by repeated inoculations with metacyclic parasites (in the order of hundreds) from infective bites during two seasons of transmission, with the consequent T cell exhaustion (22). This is quite different if compared with the probable acute stimulation due to the single intravenous injection of millions of parasites typical of an experimental infection. For this reason we believe important not only to assess the level of CICs during different stages of disease (21), but also to correlate the observed CICs levels with the time spent from the first demonstration of infection. The present study demonstrated also the usefulness of the ELISA kit for CICs detection. This test is easy to perform and does not require sophisticated instruments. A limit could be related to the generic (aspecific) assessment of CICs, independently from the pathogen that caused them or their molecular size (21). This limitation could be important, mainly in case of concomitant infectious disease(s). In our study, however, we had good evidence that the ELISA test we used for the *Leishmania* CICs detection, worked in an appropriate manner in experimental infected dogs that had no exposure to other infectious pathogens, which instead could have happened in the group of naturally-infected dogs. Ultimately, the inclusion of a laboratory infected canine model has represented a control for the ELISA assay specificity of CICs measurement in CanL. Further limitation of the present study is represented by the retrospective design that did not allow to follow further the dogs after the end of each study protocol. The detection of a low level of CICs in naïve and anti-*Leishmania* antibody negative dogs confirms that apparently aspecific immune complexes could be present in healthy individuals (23). In our case, because of the young age of the naïve beagles, we hypothesized that the low CICs amount could have been related to the core vaccinations (24).

Both symptomatic groups expressed clinical signs and clinicopathological alterations, typical of the disease. The most frequent sign related to CICs was proteinuria, that however was detected in a small proportion of sick animals, regardless the mode of infection. This indicates that proteinuria in CanL could not exclusively be related to CICs level and size, but also to other pathogenic mechanisms. The study indirectly supports the evidence that other well-known clinical immune-mediated signs such as vasculitis, uveitis and arthritis, usually observed in aged dogs affected by CanL, require longer time from the initial infection to manifest (12) than our follow up duration. In summary, this study corroborates the direct correlation between IFAT value and CICs level in the progression of the *Leishmania* infection. Nevertheless, further studies are required to determine the importance of CICs as a biomarker for diagnostic and prognostic purposes. What is clear is that CICs monitoring should be used and interpreted in different ways, depending on the status of dogs suffering from CanL. If confirmed by further studies involving more animals, CICs measurements could represent an important correlate for sick dogs at different stages of the disease; furthermore, they could be included in the preliminary biochemical panel of analyses for clinically healthy dogs found infected by *L. infantum*, in order to monitor any variations in their value during time and/or after specific treatment.

## Data Availability Statement

The datasets generated for this study are available on request to the corresponding author.

## Ethics Statement

The animal study was reviewed and approved by Veterinary Board of the Italian Ministry of Health.

## Author Contributions

MG, VF, GO, and LG contribute conception and design of the study. NB and RT organized the database. MG, VF, and GO wrote the manuscript. EF, MG, and SM performed the laboratory analyses. SM performed the statistical analysis. All authors contributed to manuscript revision, read and approved the submitted version.

## Conflict of Interest

RT was employed by the company ISOQUINEM SL. The remaining authors declare that the research was conducted in the absence of any commercial or financial relationships that could be construed as a potential conflict of interest.
